# Abnormal Levels of Serum Ferroptosis-Related Biomarkers in Diabetic Retinopathy

**DOI:** 10.1155/2022/3353740

**Published:** 2022-12-30

**Authors:** Lin Mu, Dahu Wang, Zhiguo Dong, Jiajun Wu, Xiaoyu Wu, Jing Su, Yinjian Zhang

**Affiliations:** Department of Ophthalmology, Longhua Hospital Shanghai University of Traditional Chinese Medicine, Shanghai, China

## Abstract

**Purpose:**

This study aimed to measure the concentrations of ferroptosis-related biomarkers, namely, iron (Fe), lipid peroxide (LPO), reactive oxygen species (ROS), glutathione peroxidase-4 (GPX4), and glutathione (GSH) in DR in the attempt to evaluate the diagnostic performance of these biomarkers.

**Methods:**

This study included 30 NPDR patients, 30 PDR patients, and 30 healthy subjects matched in age and sex. The concentrations of Fe, LPO, ROS, GPX4, and GSH in serum of the subjects were measured.

**Results:**

Compared with the normal group, GPX4 and GSH concentrations were significantly lower, and LPO, Fe, and ROS concentrations were significantly higher in DR patients. Compared with the PDR group, the NPDR group had higher concentrations of LPO, Fe, and ROS and lower concentrations of GPX4 and GSH, but there was no statistical difference in Fe, GPX4, and GSH. ROC curve shows that ferroptosis-related biomarkers have accumulated accuracy in NPDR and PDR.

**Conclusion:**

This study shows that ferroptosis-related biomarkers may be involved in the pathological process of DR and can be used as one of the biomarkers of DR.

## 1. Introduction

Diabetes has become the third commonest chronic disease in the world. Persistent high blood glucose damages the blood vessels in the heart, eyes, kidneys, and nerves. About 463 million patients with diabetes were reported worldwide in 2019 [1]. China has become the country with the highest incidence of diabetes [2]. Diabetic retinopathy (DR) is the most common microvascular complication of diabetes, leading to blindness in many patients.

Nonproliferative diabetic retinopathy (NPDR) is the initial stage of DR, during which microaneurysm build up and exudates emerge in the fundus. At this stage, patients may experience visual loss due to microaneurysm rupture or macular edema [3]. The end DR stage is proliferative diabetic retinopathy (PDR), during which new blood vessels generate under severe hypoxia of the fundus [4]. These new blood vessels are very fragile and easily grow into the vitreous, thus leading to severe visual impairment [5].

DR is the result of a variety of pathological processes, such as oxidative stress, mitochondrial damage, and neurodegeneration, which have played an important role in the pathogenesis of DR. Recent research has shown the critical role of ferroptosis [6–8]. Studies have shown that high glucose can increase lipid peroxide and oxidative stress levels in human retinal endothelial cells and retinal pigment epithelial cells [1, 2]. At the same time, increased lipid peroxide levels and oxidative stress were also detected in the retina of diabetic mice [3]. Ferroptosis is programmed cell death caused by iron-dependent peroxidation deposition [9]. Accumulation of iron (Fe), as well as membrane lipid peroxide (LPO), is an essential prerequisite for ferroptosis. Reactive oxygen species (ROS) also promotes the process of peroxidation. Glutathione peroxidase-4 (GPX4) is a major scavenging enzyme of LPO [10]. Glutathione (GSH) is a cofactor in the reduction of LPO by GPX4. The depletion of GSH or GPX4 is one of the important factors leading to ferroptosis [11].

Therefore, in this study, we investigated the mechanism of ferroptosis in DR by detecting the concentrations of Fe, LPO, ROS, GPX4, and GSH in serum. Our findings may provide a reference for designing new strategies for the early diagnosis and treatment of DR.

## 2. Methods

### 2.1. Subjects

The study was approved by the Ethics Committee of Longhua Hospital Shanghai University of Traditional Chinese Medicine and followed the Declaration of Helsinki (Registration number: ChiCTR2200056016). All patients volunteered to participate in the clinical study and signed an informed consent form. Thirty patients with NPDR, 30 patients with PDR, and 30 sex and age-matched healthy controls were included in this prospective clinical study. These DR patients meet the following screening criteria: Inclusion criteria include (1) type 2 diabetic patients aged between 30 and 70 whose fasting blood glucose is stable within 10 mmol/L, (2) under the clinical manifestations of NPDR and PDR, and (3) no anti-VEGF treatment within 2 months. Exclusion criteria include (1) patients with age-related maculopathy, cataracts, and other eye diseases and (2) diseases such as tumors, coronary heart disease, and Alzheimer's disease which have been reported to cause ferroptosis [4]. All subjects underwent detailed ophthalmic examinations, including best-corrected visual acuity, fundus color photography, and optical coherence tomography. Some patients underwent fundus fluorescein angiography as needed.

### 2.2. Detection of Ferroptosis-Related Biomarkers

Venous blood was drawn from all subjects and placed into ethylene diamine tetraacetic acid (EDTA) anticoagulant tubes. The blood was centrifuged at 3000 rpm for 20 min, and the supernatant was collected into a 1.5 ml Ep tube and stored at −80°C. All samples and kits were subjected to room temperature before subsequent use. Serum LPO, MDA, GPX4, and GSH were measured according to ELISA kit instructions (Shanghai Lengton Bioscience Co., China), and serum Fe was measured using a chemical method (Nanjing Jiancheng Bioengineering Institute, China).

### 2.3. Statistical Analysis

Data were statistically analyzed using SPSS 26.0 software. Measurement data were expressed as mean ± standard deviation. The Shapiro−Wilk method was used for testing the normality of measurement data; one-way analysis of the variance (LSD method was used for pairwise comparison) was used for the comparison of multiple groups of measurement data in normal distribution and with equal variance; the Kruskal−Wallis H test was used for the comparison of multiple groups of measurement data, not in normal distribution or with unequal variance (the Mann–Whitney U test was used for pairwise comparison, and Bonferroni significance was used for correction). ROC analysis is carried out with R package pROC and visualized with R package ggplot2. *P* < 0.05 was considered as statistical significance.

## 3. Results

### 3.1. Participant Characteristics

A total of 30 patients with NPDR (21 males and 9 females), 30 patients with PDR (17 males and 13 females), and 30 normal subjects (18 males and 12 females) are included in this study.  Table 1 shows the baseline characteristics of the subjects. There was no statistical difference in gender, age, diabetic macular edema (DME), and body mass index (BMI) between the three groups (*p* > 0.05). However, there was a significant difference in the duration of diabetic mellitus (DM) between the NPDR group and DR (*p* < 0.05).

### 3.2. Concentration of Ferroptosis-Related Biomarkers in NC, PDR, and NPDR Groups

**Figure 1:**
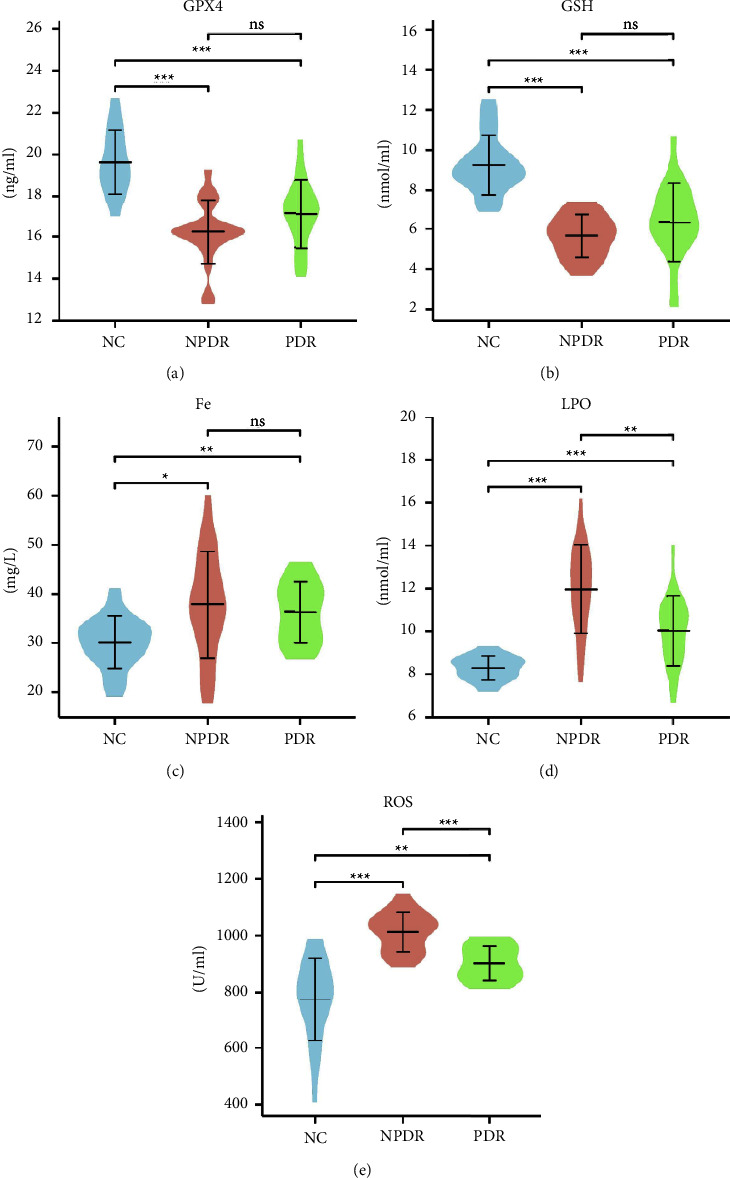
Concentration of ferroptosis-related biomarkers in NC, NPDR, and PDR groups. (a) GPX4 concentration in three groups; (b) GSH concentration in three groups; (c) LPO concentration in three groups; (d) Fe concentration in three groups; (e) ROS concentration in three groups.

As shown in Figure 1, the results showed a significant difference in each biomarker between the three groups (*p* < 0.05). GPX4 and GSH were significantly decreased (*F* = 24.896, *p* < 0.01; *F* = 30.028, *p* < 0.01), and LPO, Fe, and ROS were significantly increased (*H* = 30.132, *p* < 0.01; *H* = 10.446, *p* < 0.05; *H* = 32.697, *p* < 0.01) in DR patients. As shown in Figure 1, compared with those in the NC group, the concentrations of GPX4 and GSH were significantly lower (*p* < 0.01) and those of LPO, Fe, and ROS were significantly higher (*p* < 0.01) in the NPDR and PDR groups. Compared with the PDR group, the NPDR group had increased concentrations of LPO, Fe, and ROS (*p*=0.046, *p*=1.000, *p*=0.003, respectively) and decreased concentrations of GPX4 and GSH (*p*=0.086, *p*=0.162, respectively) but without statistical difference in Fe, GPX4, and GSH (*p* > 0.05).

### 3.3. Receiver Operating Characteristic (ROC) Analysis of Ferroptosis-Related Biomarkers

**Figure 2:**
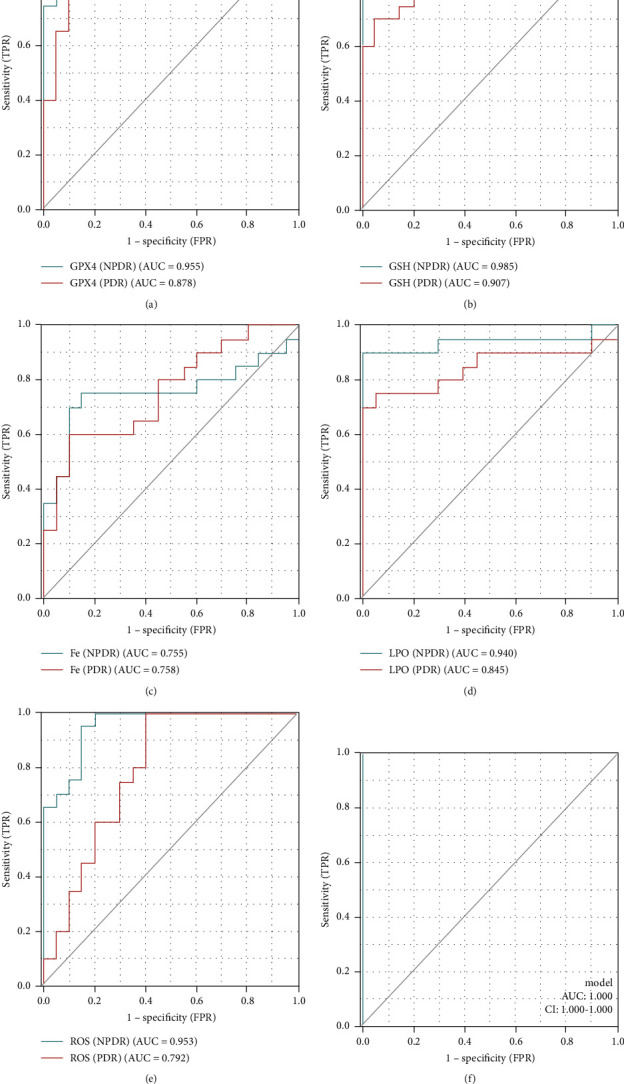
ROC curve for ferroptosis-related biomarkers in NPDR and PDR groups. (a) Diagnostic accuracy of GPX4 in NPDR and PDR; (b) diagnostic accuracy of GSH in NPDR and PDR; (c) diagnostic accuracy of LPO in NPDR and PDR; (d) diagnostic accuracy of Fe in NPDR and PDR; (e) diagnostic accuracy of ROS in NPDR and PDR; (f) diagnostic accuracy of all ferroptosis-related biomarkers in NPDR and PDR.

The ROC curves of different ferroptosis-related biomarkers are shown in Figures 2(a)–2(e). Ferroptosis-related biomarkers show good diagnostic accuracy for NPDR and PDR, and the accuracy of NPDR is higher than that of PDR. Among them, GSH has the highest accuracy (NPDR: AUC 0.985, CI 0.959–1.000%; PDR: AUC 0.907, CI 0.811–1.000%), followed by GPX4 (NPDR: AUC 0.955, CI 0.898–1.000%; PDR: AUC 0.878, CI 0.767–0.988%), followed by ROS (NPDR: AUC 0.953, CI 0.895–1.000%) PDR: AUC 0.792, CI 0.647–0.938%) and LPO (NPDR: AUC 0.940, CI 0.848–1.000%; PDR: AUC 0.845, CI 0.706–0.984%), and finally Fe (NPDR: AUC 0.755, CI 0.587–0.923%; PDR: AUC 0.758, CI 0.607–0.908%).

We performed joint indicator ROC analysis on all ferroptosis-related biomarkers. As shown in Figure 2(f), Fe, LPO, ROS, GPx4, and GSH showed significant diagnostic accuracy (AUC 1.000, CI 1.000–1.000%) for NPDR and PDR.

## 4. Discussion

In this study, we found that GPX4 and GSH were significantly lower, and LPO, Fe, and ROS were significantly higher in serum of DR patients. The results suggest that ferroptosis-related markers may play their unique roles in DR pathogenesis.

Ferroptosis, nonapoptotic regulated cell death (RCD) arising from the accumulation and peroxidation of iron, plays pathogenic roles in degenerative diseases, tumors, and ischemia-reperfusion injury [12]. The iron-dependent Fenton reaction is an essential link in the development of ferroptosis. Fenton reaction ends up with the production of toxic-free radicals and lipid peroxides [13]. Under physiological conditions, Fe bind to transferrin (TF) in serum and then migrate into cells through transferrin receptor 1 (TFR1) on the cell membrane [14]. A study shows that gene knockout of TFR1 can block this process, thereby preventing Fe poisoning caused by erastin or cystine [15]. Our study also shows a significant accumulation of Fe in DR patients.

Accumulation of LPO is a sign of ferroptosis. Polyunsaturated fatty acid-containing phospholipids (PUFA-PLs) is the main substance to be peroxidized in ferroptosis [16]. In the Fenton reaction caused by iron ions, PUFA-PLs are converted into PLOOH 14. Recent studies have shown that high glucose can increase the level of ACSL4 in ARPE-19 cells, accumulating LPO and leading to ferroptosis. In our study, the level of LPO in patients with DR was significantly higher than that in NC, which may indicate that iron death occurred in patients with DR.

ROS is the key to promote the formation of LPO [17]. In a high-glucose environment in vivo, excessive glucose is degraded through the intracellular tricarboxylic acid cycle, resulting in ROS overproduction. The retina contains large amounts of unsaturated fatty acids and is highly susceptible to being oxidized by ROS into lipid peroxides. Moreover, accumulating a large amount of intracellular ROS will inhibit the synthesis of GSH, leading to oxidative stress. Some studies have proved that oxidative stress is an essential prerequisite for mediating ferroptosis in intraocular cells. Our study found that the level of oxidative stress in patients with DR was significantly higher than that in normal patients.

The reduction of GPX4 and the inactivation of GSH play a central regulatory role in iron death aggregation. The small molecule compound RAS selective lethal 3 (RSL3), on the other hand, is able to inactivate GPX4-RSL3 to induce ferroptosis [18, 19]. Glutathione, a main antioxidant in human reduced cells, can combine with oxidative substances such as ROS to form oxidized glutathione (GSSG). GSH consists of three subunits, namely, glutamate, glycine, and cysteine. The intracellular synthesis of GSH is mainly dependent on the XC-system [20]. Erastin, on the other hand, causes insufficient cysteine synthesis by inhibiting the XC-system, which depletes GSH and ultimately induces ferroptosis [21]. Our study shows that GPX4 and GSH levels are significantly lower in DR patients. This confirms the results of existing DR studies related to ferroptosis.

To our knowledge, the present study is the first to report the levels of ferroptosis-related biomarkers in serum of DR patients. We found that the concentrations of ferroptosis-related biomarkers were more pronounced in serum of DR patients. LPO, Fe, and ROS were higher in the NPDR group than in the PDR group, but only LPO and ROS showed a statistical difference. Both GPX4 and GSH were lower in the NPDR group than in the PDR group, but without a statistical difference. The difference in the concentration of LPO and ROS between NPDR and PDR patients may be due to different pathological changes. The fundus of NPDR is mainly characterized by capillary exudation and hemorrhage, in which apoptosis of nerve cells and loss of pericytes play a significant role [1]. The formation of capillaries was the main pathological change in the PDR fundus [2]. Therefore, we speculate that ferroptosis may be involved in the apoptosis of nerve cells and pericytes, so the biological factors of iron death are more evident in NPDR. Existing studies have confirmed that diabetes can cause oxidative stress in cells of the retina and capillaries [3]. At the same time, studies have shown that oxidative stress can cause iron death in cells [4]. Therefore, the difference in the concentration of LPO and ROS between the two groups may be due to the different degrees of oxidative stress between the two groups. However, this conjecture needs to be verified by further experiments.

The current study has some limitations. First, the sample size may not be sufficient enough to verify the differences in ferroptosis-related biomarkers between NPDR and PDR. Second, only serum samples were obtained, and the profiles of these biomarkers in vitreous samples should be evaluated. Third, diabetic patients without DR are not included in this study, which may lead to inaccurate ferroptosis-related biomarkers in NPDR patients versus normal subjects.

## 5. Conclusions

In summary, ferroptosis may play an important role in DR pathogenesis. Our findings provide a probability to design new treatment strategies for DR through regulating ferroptosis. In future studies, we will conduct more comprehensive studies in larger-size groups to further verify our findings.

## Figures and Tables

**Table 1:** Baseline characteristics of the subjects.

	Control group, *n* = 30	NPDR group, *n* = 30	PDR group, *n* = 30	*P* value
Age	52.78 ± 6.45	50.74 ± 7.83	53.53 ± 8.34	0.58
Gender				0.54
Male	18 (60%)	21 (70%)	17 (57%)	
Female	12 (40%)	9 (30%)	13 (43%)	
DME		4	8	0.19
DM		5.769 ± 2.46	7.75 ± 3.73	0.04^*∗*^
BMI	20.34 ± 6.54	23.39 ± 5.46	22.34 ± 6.54	0.33

^*∗*^Statistical significance (*p* < 0.05).

## Data Availability

The data used to support the findings of this study are available from the corresponding author upon request.
